# Mindfulness-based exposure and response prevention for obsessive compulsive disorder: study protocol for a pilot randomised controlled trial

**DOI:** 10.1186/s13063-015-0664-7

**Published:** 2015-04-16

**Authors:** Clara Strauss, Claire Rosten, Mark Hayward, Laura Lea, Elizabeth Forrester, Anna-Marie Jones

**Affiliations:** School of Psychology, University of Sussex, Pevensey Building, Falmer, BN1 9QH Brighton, UK; Sussex Partnership NHS Foundation Trust, R&D Department, Sussex Education Centre, Nevill Avenue, Hove, BN3 7HZ UK; Centre for Health Research, University of Brighton, 266 Mayfield House, Village Way, Falmer, Brighton, BN1 9PH UK; Independent Consultant Clinical Psychologist, London, UK

**Keywords:** OCD, obsessive compulsive, ERP, exposure therapy, mindfulness

## Abstract

**Background:**

Obsessive Compulsive Disorder (OCD) is a distressing and debilitating condition affecting 1-2% of the population. Exposure and response prevention (ERP) is a behaviour therapy for OCD with the strongest evidence for effectiveness of any psychological therapy for the condition. Even so, only about half of people offered ERP show recovery after the therapy. An important reason for ERP failure is that about 25% of people drop out early, and even for those who continue with the therapy, many do not regularly engage in ERP tasks, an essential element of ERP. A mindfulness-based approach has the potential to reduce drop-out from ERP and to improve ERP task engagement with an emphasis on accepting difficult thoughts, feelings and bodily sessions and on becoming more aware of urges, rather than automatically acting on them.

**Methods/Design:**

This is a pilot randomised controlled trial of mindfulness-based ERP (MB-ERP) with the aim of establishing parameters for a definitive trial. Forty participants diagnosed with OCD will be allocated at random to a 10-session ERP group or to a 10-session MB-ERP group. Primary outcomes are OCD symptom severity and therapy engagement. Secondary outcomes are depressive symptom severity, wellbeing and obsessive-compulsive beliefs. A semi-structured interview with participants will guide understanding of change processes.

**Discussion:**

Findings from this pilot study will inform future research in this area, and if effect sizes on primary outcomes are in favour of MB-ERP in comparison to ERP, funding for a definitive trial will be sought.

**Trial registration:**

Current Controlled Trials registration number ISRCTN52684820. Registered on 30 January 2014.

## Background

Obsessive compulsive disorder (OCD) is a distressing and debilitating mental health condition affecting approximately 1 to 2% of the population [1,2]. People with OCD experience unpleasant, repetitive, unwanted and intrusive thoughts (for example, thoughts of harm coming to a loved one) and engage in compulsive behaviours that are meaningfully related to the thought in terms of preventing harm (for example, repeated checking or cleaning) or that are intended to reduce anxiety.

The behavioural theory of OCD draws on behavioural theory of anxiety disorders more broadly and suggests that compulsive behaviours are maintained through the process of negative reinforcement. That is, compulsive behaviours result in the temporary relief of anxiety, and therefore, these behaviours are reinforced, becoming more likely to occur in the future [3]. This theory informed behavioural therapy for OCD: Exposure and Response Prevention (ERP) [4]. The therapy encourages people to gradually and regularly expose themselves to triggers of their intrusive thoughts (for example, touching surfaces perceived as ‘contaminated’) whilst not engaging in their usual compulsive behaviours (for example, hand washing). Although ERP was developed in the 1960s [4], it is still the form of psychological therapy for OCD with the most robust evidence of effectiveness. Consequently, national treatment guidelines in the UK recommend ERP as *the* psychological therapy for OCD [2]. The guidelines recommend either individual or group-based ERP, and there is randomised controlled trial evidence that group ERP is effective [5] and that individually delivered cognitive behavioural therapy for OCD is not more effective than when delivered in a group [6].

Despite the success of ERP, there are substantial limitations. Only about half of people with OCD meet recovery criteria after a course of ERP [7]. Primarily, this seems to be because many people find ERP too challenging; by definition, the therapy involves intentionally and regularly exposing oneself to anxiety-provoking situations and disengaging from efforts to eliminate anxiety. Twenty-five percent of people drop out of therapy early [8]. Moreover, people with OCD show poor distress tolerance [9] and are therefore particularly likely to find it difficult to engage in exposure-based therapies. Even among those who do complete a course of ERP, many do not fully engage with the regular between-session exposure and response prevention tasks [10], with lower rates of task engagement being associated with poorer therapy outcomes [10]. Therefore, there is a real need to find ways of making ERP more acceptable to patients in order to reduce drop-out rates and increase engagement with exposure tasks. Increasing patient engagement in this way will hopefully lead to a greater number of patients meeting recovery criteria upon completion of an ERP course of treatment.

Mindfulness is a state of awareness characterised by non-judgemental, accepting attention towards current experiences, such as thoughts, feelings and bodily sensations. Training in mindfulness has been incorporated into mindfulness-based interventions (MBIs) in recent years [11,12]. There is increasing evidence that MBIs have positive consequences for psychological [13,14] and physical [15] health, in a broad range of mental health [16-18] and nonclinical [19,20] populations.

Despite this wealth of literature, there is a paucity of research of MBIs for OCD [21]. However, a mindfulness-based approach might be expected to enhance engagement with ERP for three reasons. First, exposure during ERP elicits intrusive thoughts that the person would typically attempt to eliminate by engaging in compulsive behaviours. It has long been established that intrusive thoughts are common throughout the general population and are not usually problematic [22]. Mindfulness-based interventions teach people to allow such thoughts into awareness without attempting to suppress them and to attend to them non-judgementally [12]. Therefore, a mindfulness-based approach to ERP would be expected to enable people to be better able to accept the intrusive thoughts elicited following exposure to triggering situations and to remain engaged in the ERP task despite these thoughts. Second, it is also well established that people with OCD show a heightened intolerance of anxiety [9]. Just as with thoughts, MBIs teach people to notice and accept unpleasant physical sensations and anxiety and to disengage from attempts to avoid or eliminate them [12]. It would be expected, therefore, that a mindfulness-based approach would help people to attend to and accept the physical sensations of anxiety that come about during ERP tasks and to, nevertheless, remain engaged with the tasks. Third, MBIs encourage people to notice the range of behavioural choices they can make in response to an event, rather than reacting to such events automatically [12]. A mindfulness-based approach to ERP should, therefore, support people to better recognise their urges to engage in compulsive behaviours and to make the choice to resist these urges during ERP tasks. Mindfulness-based interventions are usually offered in groups, and ERP can be offered either individually or in groups [2]; thus, a group approach to integrating MBI with ERP is warranted.

A group of six people, all with lived experience of OCD who had experience of ERP and mindfulness-based approaches, were consulted when developing this protocol. The group advised that an integrated mindfulness-based ERP group would be more likely to improve symptom outcomes and to enhance engagement in comparison to either type of intervention (that is, MBI or ERP) on its own. In light of this advice and the background literature outlined above, we expect that a mindfulness-based approach to group ERP would be more effective and have lower rates of drop-out than group ERP on its own.

Given the lack of research in this area to date [21], this is a protocol for a pilot randomised controlled trial (RCT). The research question for the definitive trial is as follows: ‘Is group mindfulness-based ERP more effective at reducing OCD symptom severity and better at enhancing therapy engagement than standard group ERP for people diagnosed with OCD?’ The primary aim of this pilot study is to estimate the size and direction of the treatment effect, and the corresponding 95% confidence interval, by comparing mindfulness-based ERP (MB-ERP) groups to standard exposure and response prevention (ERP) groups on the primary outcome measures of OCD symptom severity and therapy engagement. If the magnitude of these effects are deemed clinically relevant, then the estimate will be used in a power calculation for the definitive trial. Secondary aims of the definitive study will be to test effects on other important outcome (depression and wellbeing) and process (mindfulness and obsessive-compulsive beliefs) measures and so these measures are included in the pilot study to assess their feasibility. In addition, the pilot study will gather information on rates of recruitment and will record rates of attrition from the study. A semi-structured interview with participants will also be conducted to ascertain experiences of change and attributions for change from participants’ perspectives. Information from these interviews will inform the development of future research in this area.

## Methods/Design

### Design and sample size

This is a pilot study for a single blind, prospective RCT using an intention-to-treat comparison of two treatment groups (MB-ERP and ERP). Measures will be taken at baseline (Time 1), post-therapy (Time 2) and at 6 months post-therapy (Time 3). A power calculation to determine sample size is not appropriate for this pilot study [23,24], as there is no intention to identify a statistically significant difference between the two treatment groups. This study follows recommendations for pilot RCTs [25] and aims to have at least 12 participants per treatment arm who provide full data. We aim to recruit 40 people with OCD to allow up to 40% attrition from the study, which is a conservative estimate of what might be expected to occur in ERP [8].

This study has received full ethical approval through the South East Coast (Surrey) arm of the National Research Ethics System in the UK (Research Ethics Committee reference: 13/LO/1768).

### Participants

Participants will be 40 adults referred to a mental health trust in the South of the England. Inclusion criteria are that participants (1) meet DSM-IV [26] diagnostic criteria for OCD; (2) have been stable on psychiatric medication for at least 3 months prior to the consent meeting; (3) have no plans for changes to psychiatric medication during the course of the study; (4) have not received psychological therapy in the past three months or have any plans for psychological therapy during the course of the study; and (5) are older than 18 18 years of age. Exclusion criteria are as follows: those who have an identified organic cause for their OCD symptoms, a diagnosed learning disability, or if they meet the diagnostic criteria, based on the Mini International Neuropsychiatric Interview (MINI version 6.0.0) [27], for a psychotic disorder, post-traumatic stress disorder, anorexia nervosa, alcohol abuse or substance abuse (non-alcohol). This will be ascertained through the care team. To reflect the reality of mental health services and the comorbidity of OCD with other mental health conditions [2], comorbidity will not be an exclusion criterion. People presenting with hoarding-only compulsions will be excluded from the study, given the recent move to classify hoarding as distinct from OCD [1].

### Measures

#### Diagnostic status

*Mini International Neuropsychiatric Interview* (*MINI version 6.0.0*) [27]. DSM-IV OCD diagnostic criteria [26] will be established at all three time points using the Mini International Neuropsychiatric Interview. Meeting diagnostic criteria at baseline is an inclusion criterion for the study.

#### Primary outcome measures

*Yale-Brown Obsessive Compulsive Scale - Second Edition (Y-BOCS-II)* (Goodman, Rasmussen, Price & Storch: Yale-Brown Obsessive Compulsive Scale – Second Edition Manual, unpublished). This measure is the primary outcome measure, rather than OCD diagnostic status, as symptom severity is a continuous variable and is, therefore, more informative about changes to OCD symptom severity. The Y-BOCS-II is considered the gold standard measure of OCD symptom severity [28]. It has excellent indices of reliability and validity, including internal consistency and test re-test reliability alpha coefficients of over 0.8, and strong correlations with clinician measures of OCD severity [28].

##### Exposure and response prevention engagement

Engagement will be measured in two ways: (1) the number of therapy sessions attended (maximum = 10) will be recorded, and (2) participants will be asked to record in a daily diary the number of ERP tasks performed each day.

#### Secondary outcome measures

*Short Warwick-Edinburgh Mental Well-Being Scale* [29]. The short version of the Warwick-Edinburgh Mental Well-Being Scale is a 7-item measure of well-being. Stewart-Brown and colleagues [30] reported strong internal consistency, test re-test reliability, and concurrent validity and found that the measure is sensitive to change in mental health populations.

*Beck Depression Inventory - second edition (BDI-II)* [31]. Depression is often comorbid with OCD, with depression thought to arise as the secondary condition [2]. The BDI-II is one of the most widely used measures of depressive symptoms. Beck and colleague [31] reported excellent internal consistency and test re-test reliability (α >0.9 for both). Concurrent validity with the Hamilton Psychiatric Rating Scale for Depression-Revised is also good (r = 0.71).

*Five-Facet Mindfulness Questionnaire - Short Form (FFMQ-SF)* [32]. The FFMQ-SF is a 24-item self-report scale assessing five mindfulness facets: observing, describing, acting with awareness, non-judgement and non-reacting. The short form has been reported to have adequate indices of reliability (α > .73 for each subscale) and validity [32].

*Obsessional Beliefs Questionnaire - Revised (OBQ-44)* [33]. The OBQ-44 is a 44-item self-report measure of cognitions associated with OCD. The instrument has three subscales: (1) Responsibility/Threat Estimation, (2) Perfectionism/Certainty, and (3) Importance/Control of thoughts. The scales have excellent internal consistency (α > .89 for each subscale), and the total score on the OBQ-44 distinguishes between people diagnosed with OCD and non-OCD anxious controls [33].

*Change Interview* [34]. The Change Interview is a semi-structured questionnaire designed to ask participants their experiences of a psychological intervention. Specifically, it asks about changes that have occurred in the person’s life since starting the intervention and to what they attribute these changes. Changes can be attributed to the intervention or to other factors. Finally, participants are asked to comment on the aspects of the intervention that helped change to occur and those aspects that might have hindered change from occurring.

### Procedure

**Figure 1:**
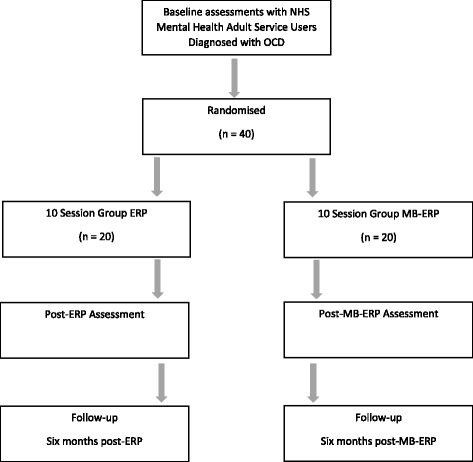
CONSORT diagram. ERP, exposure and response prevention; MB-ERP, mindfulness-based exposure and response prevention; NHS, National Health Service; OCD, obsessive compulsive disorder.

Figure 1 shows the flow of participants through the pilot RCT. Patients with a diagnosis of OCD will be sought through mental health teams in the host NHS trust. Informed consent will be obtained from each participant. Potential participants will be given a copy of the study participant information sheet and will have the opportunity to discuss the study in person with the research assistant (RA) before signing the consent form. The study research assistant will conduct the baseline assessments of consenting participants on the above measures within four weeks of the groups starting. After baseline assessments have been completed, participants will be randomly allocated to either an MB-ERP or an ERP group by a Clinical Trials Unit using block randomisation. Ten participants will be allocated to each group so that there will be two MB-ERP groups and two ERP groups in total. Ten therapy sessions for each group will be facilitated by two clinical psychologists, at least one of whom will be an accredited Cognitive Behavioural Therapy (CBT) therapist and an accredited mindfulness-based cognitive therapy teacher. Sessions will be held either in NHS premises or suitable community venues. Post-group assessments on each of the outcome measures will be conducted by a second RA who will be blind to group allocation.

### Therapy protocol

#### Exposure and response prevention groups

Participants in the ERP condition will be provided with 10 sessions of group ERP, with each session lasting two hours. The group protocol is adapted from a manual (Van Noppen, Stekette & Pato: Group Behaviour Therapy (GBT) Treatment Manual for Obsessive Compulsive Disorder (OCD), unpublished). Session 1 introduces the rationale for ERP. Sessions 2 to 9 involve participants designing ERP tasks. Rather than developing graded hierarchies, participants will design ERP tasks that are expected to elicit the greatest levels of anxiety they are willing to tolerate in relation to specific OCD areas of difficulty [35]. One or more ERP task will be conducted during the therapy session, with encouragement and support offered by a group facilitator. During in-session ERP tasks, ratings of anxiety will be recorded at regular intervals for each participant on a whiteboard in order to observe the rise and fall in anxiety during the task. Following this, each participant will be asked to identify three ERP tasks to conduct each day between therapy sessions and to record daily engagement in these tasks using a diary. In addition, participants will be encouraged to engage in unplanned ERP tasks in daily life when facing obsessional cues, that is, to take as many opportunities to apply the ERP approach to OCD as possible, and this will include both planned ERP weekly tasks and unplanned ERP tasks when the opportunity arises [35]. Session 10 will focus on consolidating learning from the therapy group and developing a therapy blueprint for continuing ERP once the group has ended. Regular supervision will be provided for the group facilitators by an ERP expert who will also rate and provide feedback on audio recorded therapy sessions to monitor therapy fidelity.

#### Mindfulness-based exposure and response groups

The MB-ERP group will be provided with 10 sessions of mindfulness-based behaviour therapy (ERP), each session lasting two hours. Session 1 will introduce the rationale for ERP alongside the rationale for including mindfulness principles and practice. Sessions 2 to 9 will each start with a 10-minute mindfulness practice: mindfulness of the breath and body (session 1); mindfulness of the breath, body, sounds and thoughts (sessions 2-3); mindfulness of thoughts (session 4-5); and mindfulness of body, thoughts, urges and action (sessions 6-10). Mindfulness practice will be followed by a 20-minute Socratic inquiry about the practices in order to draw out learning, with a particular emphasis on the three areas noted above: non-judgmental acceptance of (intrusive) thoughts, non-judgmental acceptance of physical sensations (of anxiety) and a recognition that it is possible to choose whether or not to follow behavioural urges (to engage in compulsive behaviours). A three-minute mindfulness breathing space practice [12] will also be introduced from session 6 onwards for participants to use on a daily basis to bring awareness to current experiences and to facilitate engagement in ERP tasks. In session 1, participants will be given an MP3 player with the range of mindfulness practices for daily home practice, with practice being recorded in the daily diary. The final 90 minutes of MB-ERP session will follow the same ERP protocol outlined above with the addition that whilst engaging in ERP tasks, participants will be encouraged to bring mindfulness skills to the tasks. In particular, this will involve an encouragement to bring mindfulness to intrusive thoughts as they arise (allowing thoughts to arise without suppressing them or becoming absorbed with them), to bring mindfulness to physical sensations of anxiety (noticing and accepting physical sensations of anxiety with curiosity and interest) and to bring mindfulness to urges to engage in compulsive behaviours (noticing compulsive urges and recognising there is a choice available - a choice to follow the urge and a choice to allow the urge to remain, without following it). Session 10 will focus on consolidating learning from the therapy and developing a therapy blueprint for continuing MB-ERP after the therapy has concluded. As with the standard ERP groups, therapy sessions will be audio recorded and rated by an expert to monitor and ensure therapy fidelity. This expert will provide regular supervision to the group facilitators. Additional supervision will be provided by an expert MBI therapist in order to ensure adherence to MBI principles and practice.

### Data analysis

Participant characteristics will be examined at baseline to check that the randomisation procedure has been successful and any differences between the two treatment groups are at a minimum. This will include a check of between-group differences on demographic variables (age and gender) and on all the outcome measures by examining the descriptive statistics. The primary outcomes will be evaluated through an intention-to-treat analysis using linear mixed models to allow for missing data that is assumed to be missing at random. For each primary outcome, treatment effects will be calculated simultaneously at both the post-therapy and the six-month follow-up time points after adjusting for the outcome measure at baseline and site. The models will include treatment group (ERP or MB-ERP), time (post-therapy or six-months follow-up) a time-treatment interaction and a random intercept. Corresponding standard errors, significance levels and 95% confidence intervals will be calculated for all treatment effects.

Data from the Change Interview will be analysed using Thematic Analysis [36] within each condition (that is, a separate thematic analysis for the ERP and MB-ERP conditions). This will elucidate participants’ subjective experiences of change in each type of intervention and participants’ views on mechanisms of change, which the Change Interview specifically addresses.

For the full trial, effect sizes alongside the minimum clinically important difference, where relevant, on the primary outcomes (Y-BOCS-II and engagement) will be used to conduct a sample size calculation. Recruitment rates to the study will be presented in terms of ‘number of participants consented to the study per research assistant days - that is, how many research assistant days are taken on average in order to consent one participant. This will allow us to estimate the research assistant time that will be needed for recruitment in a full trial, once the sample size calculation is conducted. Data completeness will be monitored, and the percentage of consenting participants contributing to all data-collection points will be reported. If completeness falls below 95%, the research team will consider strategies for improving future data collection and handling missing data. Similarly, if once recruited, the retention rate falls below 80%, the research team will explore methods to increase retention in the full trial. Data quality will be overseen by the trial statistician.

Between-group effect sizes and data from the Change Interview will inform a funding application for a definitive trial of MB-ERP. If effect sizes on the primary outcome measures (Y-BOCS-II, number of therapy sessions attended and number of between-session ERP tasks completed) are in favour of MB-ERP in comparison to ERP, funding for a definitive trial will be sought, which may include modifications to the current therapy and study design depending on findings from the quantitative data analysis, review of recruitment and retention rates, and from the Change Interview. If effect sizes on the primary outcome measures are not in favour of MB-ERP in comparison to ERP, the research team will review possible reasons for this, drawing on the Change Interview findings and this will inform the next stage in this programme of research.

## Discussion

Exposure and Response Prevention (ERP) is the most well-established and strongly evidenced psychological therapy for obsessive compulsive disorder (OCD). However, the approach is challenging for patients because it involves them directly and regularly confronting their anxiety-provoking triggers and staying with their feelings of anxiety, rather than attempting to eliminate them by engaging in compulsive behaviours. It is perhaps not surprising, therefore, that many people refuse ERP as a treatment option [37], that about a quarter of people who do start ERP drop out [8] and that many of those who do remain engaged in therapy sessions fail to engage in the recommended regular between-session ERP tasks, which is associated with poorer therapy outcomes [10]. Taken together, around half of people with OCD fail to show recovery after ERP [7].

Mindfulness-based ERP (MB-ERP) for OCD has the potential to increase engagement with ERP and, therefore, could potentially improve therapy outcomes. The therapy encourages patients to attend non-judgementally to their intrusive thoughts, increase their acceptance of physical sensations of anxiety, and bring greater awareness to urges to engage in compulsive behaviours. This pilot study directly compares MB-ERP groups to standard ERP groups with the primary aim of generating effect sizes for OCD symptom severity and measures of therapy engagement in order to calculate the sample size needed for a definitive trial. The hypothesis for the definitive trial is that, in comparison to ERP, MB-ERP will lead to a greater reduction in OCD symptom severity and greater rates of participant engagement.

Exposure and Response Prevention has remained relatively unchanged since its development almost 50 years ago and yet it remains the most effective therapy for OCD [2]. Mindfulness-based ERP has the potential to enhance the effectiveness of ERP through improving participant engagement in therapy sessions and in therapy tasks. This pilot study is the first step in evaluating this possibility and the next steps will be determined by the outcomes of this study.

## Trial status

At the time of manuscript submission, recruitment for this study was ongoing.

## Abbreviations

DSM-IV: The Diagnostic and Statistical Manual of Mental Disorders, fourth edition
ERP: exposure and response prevention
MB-ERP: mindfulness-based exposure and response prevention
MBI: mindfulness-based interventions
OCD: obsessive compulsive disorder
RCT: randomised controlled trial
